# Focusing on the Differences of Resting-State Brain Networks, Using a Data-Driven Approach to Explore the Functional Neuroimaging Characteristics of Extraversion Trait

**DOI:** 10.3389/fnins.2018.00109

**Published:** 2018-03-05

**Authors:** Feng Tian, Junjie Wang, Cheng Xu, Hong Li, Xin Ma

**Affiliations:** ^1^Beijing Anding Hospital of Capital Medical University, Beijing, China; ^2^Department of Psychiatry, The Second Hospital of Shanxi Medical University, Taiyuan, China; ^3^Department of Psychiatry, First Hospital/First Clinical Medical College of Shanxi Medical University, Taiyuan, China; ^4^Department of Magnetic Resonance Imaging, Shanxi Province People's Hospital, Taiyuan, China; ^5^Institute of Psychology, Chinese Academy of Sciences, Beijing, China

**Keywords:** personality traits, resting-state fMRI, data-driven, salience network, extraversion trait

## Abstract

In recent years, functional magnetic resonance imaging (fMRI) has been widely used in studies that explored the personality-brain association. Researches on personality neuroscience have the potential to provide personality psychology with explanatory models—that is, *why* people differ from each other rather than *how* they differ from each other (DeYoung and Gray, 2009). As one of the most important dimensions of personality traits, extraversion is the most stable core and a universal component in personality theory. The aim of the present study was to employ a fully data-driven approach to study the brain mechanism of extraversion in a sample of 111 healthy adults. The Eysenck Personality Questionnaire (EPQ) was used to measure the personality characteristics of all the subjects. We investigated whether the subjects can be grouped into highly homogeneous communities according to the characteristics of their intrinsic connectivity networks (ICNs). The resultant subjects communities and the representative characteristics of ICNs were then associated to personality concepts. Finally, we found one ICN (salience network) whose subject community profiles exhibited significant associations with Extraversion trait.

## Introduction

As a core concept of psychology, the study of personality is the basis of the practical application of psychology. Since the patterns of behavior and cognition that constitute personality tend to be stable and broadly predictable (Canli and Amin, 2002; DeYoung and Gray, 2009), it is feasible for us to uncovering how personality is encoded in the brain from the perspective of cognitive neuroscience. In recent years, a growing number of studies using functional magnetic resonance imaging (fMRI) have explored the personality-brain association (Ryan et al., 2011; Wang et al., 2014; Wei et al., 2014; Cohn et al., 2015). However, previous studies were usually driven by personality concepts formed by observer-dependent life experience and consensus (Kunisato et al., 2011; Wei et al., 2011). Whether the concepts that are not completely independent of the observer can objectively reflect the functional organization of the brain networks is questionable. As pointed out by Jonathan et al. (Adelstein et al., 2011) “The large-scale and data-driven methods should be incorporated into future studies in the aim of examine the neural correlations of personality more comprehensively” (Kunisato et al., 2011).

More recently, there is an increasing number of studies using independent components analysis (ICA)—a data-driven approach, to explore the intrinsic connectivity networks (ICNs) from resting-state fMRI data of multiple subjects (Frazier et al., 1996, 1997; Hollis, 2000) with an assumption that the group is homogeneous and all the subjects share common components. However, this assumption effectively ignores the ample evidence for the heterogeneous nature of brain. In the field of clinical psychiatric diagnosis, a growing number of studies have explored the subtypes of neuropsychiatric disorders according to the heterogeneous nature of brain diseases (Fair et al., 2012, 2013; Drysdale et al., 2017; Gupta et al., 2017; Varol et al., 2017). Besides, as mentioned before, since the personality concept was not designed to facilitate people biological differentiation, there is a high potential that subjects in the groups divided based on personality inventory might be highly heterogeneous in brain mechanism (Di Martino et al., 2009; Adelstein et al., 2011). Thus, against simply comparing ICNs between pre-defined subject groups under the assumption that subjects belong to the same concepts-based group share homogeneous ICNs characteristics, we decide adopt a systematic data-mining approach, generalized ranking and averaging independent component analysis by reproducibility (gRAICAR) (Yang et al., 2008, 2012, 2014a,b), which allows us to classify all subjects into different communities by taking into account information from inter-subject variability, to find the behavior-based personality traits consistent with the biological classification.

**Figure 1:**
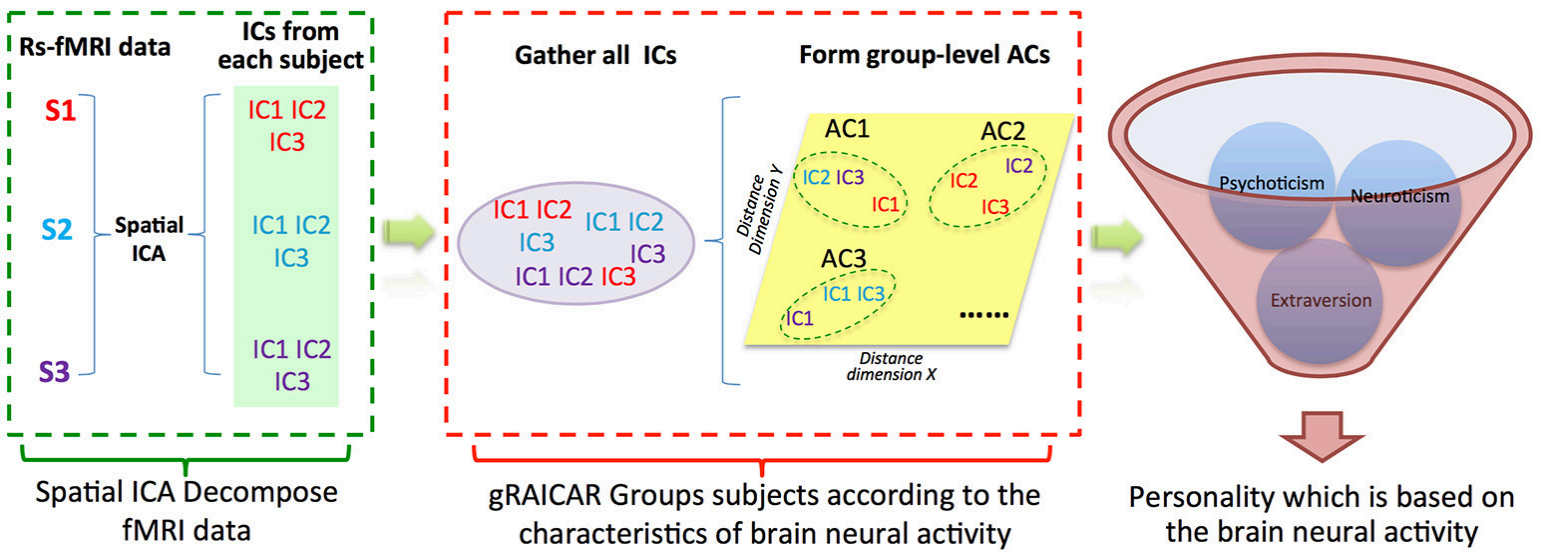
Demonstration of data analysis flow. For simplicity, we assume that there are only three subjects (denoted as S1, S2, and S3). First, the fMRI data of subjects are decomposed individually by using spatial independent component analysis (ICA) into spatial components (ICs). Assume that for each subject we can get three ICs that are color coded to indicate which subject they are from. The resultant ICs maps are presented in the green layer. Second, all of the ICs from individual subjects were pooled in gRAICAR (as presented in the purple layer). We present a distance space depicting the similarity between all ICs in the yellow layer. The intention of gRAICAR in this part is to identify ICs that are from different individuals but are close to each other (as marked with green dashed circles). The group-level aligned components (ACs) were formed by these clustered ICs sequentially, and a community detection algorithm can be applied to each AC to identify homogeneous subject communities among all subjects. Third, we try to seek a kind of personality trait, according to which the subjects could be grouped into communities that maximally agreed with the brain neural activity derived communities.

In the present study, we recruited 111 subjects to investigate whether the subjects can be grouped into communities according to the characteristics of their ICNs measured using resting-state fMRI. After that, we associated the Eysenck Personality Questionnaire (EPQ) (Eysenck and Eysenck, 1975) to the ICN-derived subject communities to explain the findings in neuroimaging data, aiming to find the potential one-to-one mapping between ICNs and each traits of EPQ (see Figure 1 for a graphical demonstration of data analysis process). Finally, we found one ICN (salience network) whose subject community profiles exhibited significant associations with EPQ-Extraversion trait.

## Methods

### Participants

All participants were recruited from Shanxi medical university or advertisements on bulletin boards in the community. The inclusion criteria were: (1) age > 18 years, (2) right handedness, (3) no history of psychiatric disorders or neurological disease, brain injury. The exclusion criteria were: (1) history of substance abuse (including illicit drugs and alcohol), (2) unsuitability for MRI scans (metal implants or claustrophobia). Finally, 111 subjects (age range: 19–60 years; 50 males, see Table 1 for details) are used for subsequent image analyses. The study protocol was approved by the Ethics Committee of Shanxi Medical University. Written informed consents were obtained from all participants. After the participants completed all tests, they subsequently received payment for their time.

**Table 1:** The descriptive statistics for the demographic characteristics of sample.

	**Age range**	**Male**	**Female**	**Number**	**Percentage**
Sample (111)	18–20	0	2	2	1.80
	21–30	16	16	32	28.83
	31–40	11	9	20	18.02
	41–50	16	25	41	36.93
	51–60	8	8	16	14.42

### Behavior data acquisition

We collected personality characteristics of all subjects by using EPQ, an 88-item true-false self-report scale, which has been widely used in China and has been proved validated (Chen et al., 2016).

### MRI data acquisition

All MRI data were acquired on a 3.0 T SIEMENS Trio scanner at Shanxi Provincial People's Hospital. Each participant completed a 8′50′ resting-state fMRI scan using an echo-planar imaging (EPI) sequence (32 axial slices, acquired from inferior to superior in an interleaved manner, FOV = 240 mm, matrix = 64 × 64, slice thickness = 4.0 mm, gap = 0.0 mm, TR/TE = 2,500/30 ms, FA = 90°, 212 volumes). Anatomical scans were also acquired for each participant using a T1-weighted 3D MP-RAGE sequence (160 continuous sagittal slices, slice thickness = 1.2 mm, FOV = 225 × 240 mm, matrix = 240 × 256, TR/TE/TI = 2,300/2.95/900 ms, FA = 9°). All Subjects were instructed to close their eyes and remain awake during the scan. After the scans, all subjects confirmed that they did not fall asleep during the scan.

### T1 image preprocessing

The images were preprocessed using the Connectome Computation System (CCS:http://zuolab.psych.ac.cn/ccs.html) (Zuo et al., 2013),-an integration system that involves AFNI, FSL, Freesurfer (Cox, 2012; Fischl, 2012; Jenkinson et al., 2012). Individual T1 images preprocessing primarily include: (1) the images were processed with a spatially adaptive non-local mean filter (Zuo and Xing, 2011), and submitted into the *recon-all* routine in FreeSurfer 5.1 to extract the brain tissues, (2) all individual anatomical brain images were transformed into the MNI152 standard space by using Advanced Normalization Tools (ANTs, http://stnava.github.io/ANTs/) (Avants and Gee, 2004).

### Resting-state fMRI preprocessing

The functional image preprocessing included: (1) discarding the first five EPI volumes from each scan to allow for MRI signal equilibration, (2) correcting for slice timing difference, (3) correcting for rigid head motion, (4) estimating a rigid transformation from individual functional space to the corresponding anatomical space by using ANTs, (5) normalizing the 4D data to a global mean-based intensity of 10,000, (6) band-pass filtering (0.01–0.1 Hz). Finally, the preprocessed data were used in individual-level ICA analyses in gRAICAR.

To ensure the data were usable for subsequent analyses, a data quality control procedure (QCP) was conducted. The structural images were visually inspected by two researchers for the quality of tissue segmentation and brain registration. For functional images, subjects were excluded for excessive head motion, as measured by the root-mean-square of frame-wise displacement larger than 0.2 mm.

### gRAICAR network mining analysis

The algorithm of gRAICAR was applied to the preprocessed functional images for the purpose of characterizing the consistency of the ICNs, in other words, finding a one-to-one correspondence between component maps across all of the subjects. If the activity sources in the data are similar, the similar spatial patterns in component maps should be detected by ICA. Thus, the method we employed in this study could tell us how strong the one-to-one correspondence is, subsequently, we could reveal variations of brain maps across different subjects. The rationale and a more detailed illustration of gRAICAR algorithm have been described in the original (Yang et al., 2012) and morerecent paper (Yang et al., 2014a,b). We also computed the Ratio of Significant Subjects (RSS) within all subjects. The change of this value across all subjects reflected the trend in fractions of subjects who possess the given network.

## Results

gRAICAR identified 30 ACs. Based on their spatial patterns and on previous literature (Beckmann et al., 2005; Damoiseaux et al., 2006, 2008), 12 ACs were found to represent functional ICNs. The remaining 18 components reflected artifacts like movement, cerebrospinal fluid flow, and physiological noise.

The inter-subject similarity matrix for each of the 12 ICNs reflected a subject community profile which reflect potential subgroups of subjects that share similar ICN characteristics. Thus, we combined with the personality score for each subject, through the permutation test and visual inspection, trying to find which original confused inter-subject similarity matrix becomes a regular distribution then that means this ICN-derived subject community may be related to personality classification. In the following sections, we report one ICN-derived subject community that are most related to EPQ-Extraversion classification.

### Behavioral results

The demographical information and descriptive statistics for the EPQ scores of all participants are shown in the Tables 1, 2.

**Table 2:** The descriptive statistics for the EPQ scores of all participants in the sample.

**Dimension of EPQ**	**Mean**	***SD***	**Range**
Neuroticism	46.80	10.09	25–75
Extraversion	56.22	10.36	35–80
Psychoticism	48.74	9.76	35–85

### One ICN associated with EPQ-extraversion classification

**Figure 2:**
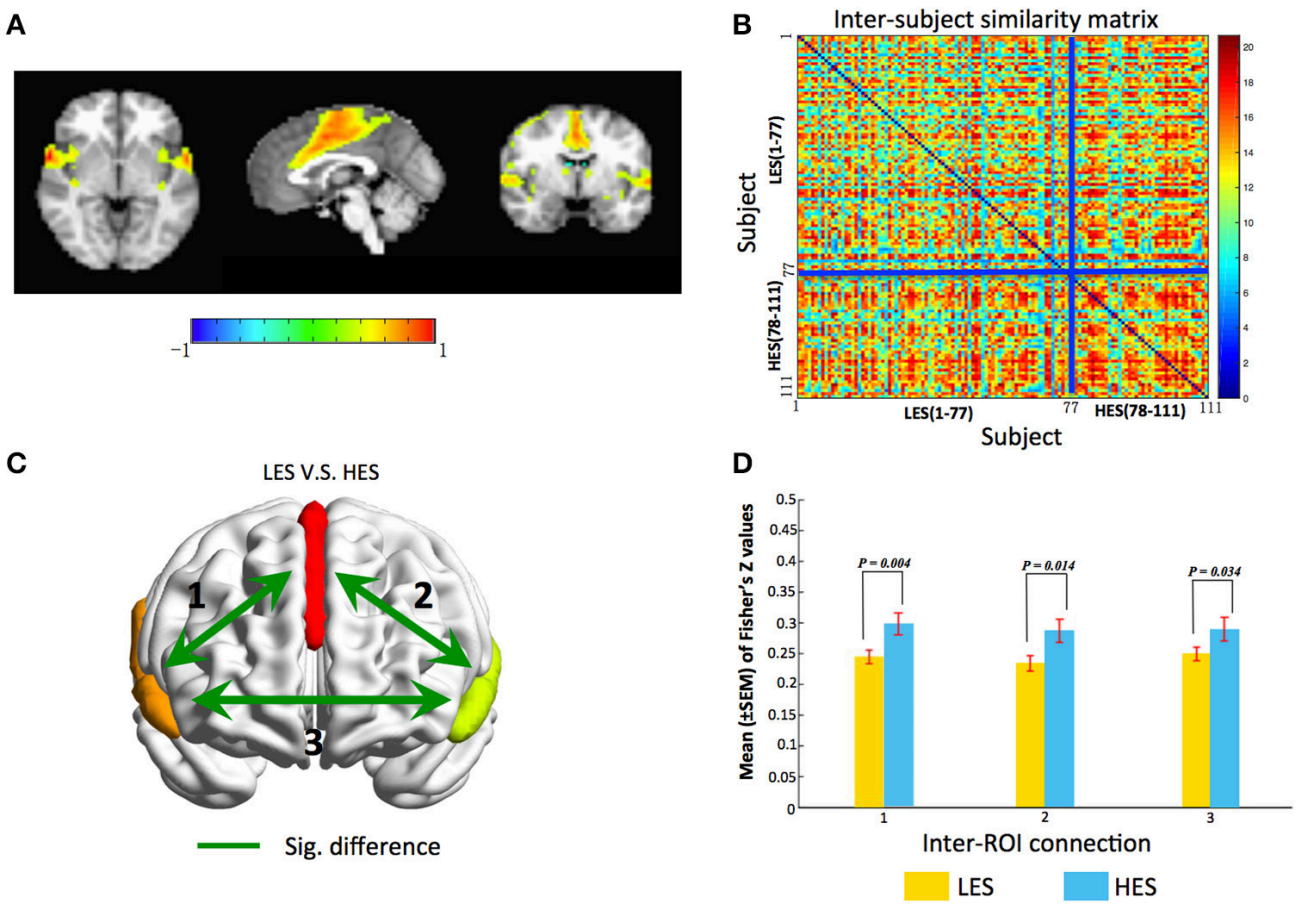
gRAICAR reveal the salience network is associated with Extraversion classification. **(A)** The salience network dominated by the anterior cingulate and bilateral anterior insula. **(B)** Combined with the Extraversion scores, the similarity matrix change into a regular distribution. Compared to the LES subjects, the HES subjects have a higher inter-subjects average similarity. For visualization purpose, the subjects are grouped into LES and HES groups, and the blue solid lines mark the boundary between the two groups. **(C)** Map of the salience network showing inter-regional connections exhibiting significant (green line) differences in connectivity strength (Fisher's Z) between LES and HES groups. **(D)** Bar graphs showing separately statistical details comparing functional connectivity strength between LES and HES groups. Labels along the horizontal axis correspond to the connections marked on **(C)**. All three inter-ROI connections show significant difference between LES and HES groups. The surface maps are rendered in BrainNet Viewer (Xia et al., 2013).

We found one ICN, that is, salience network (SN) comprising bilateral insula and anterior cingulate cortex (ACC) reflected a subject community profile associated with the EPQ-Extraversion classification (Figure 2). The inter-subject similarity matrix of this SN depicts similarities within all of the subjects. We have added a permutation test of which the process is as follows. First, the difference in the average similarity degree between the high extroversion score (HES) subjects (*n* = 34) and the low extroversion score (LES) subjects (*n* = 77) was calculated. Then the HES subjects and the LES subjects were pooled. Next, the subjects were randomly sampled with replacement while keeping the original sample sizes for the HES subjects (*n* = 34) and the LES subjects (*n* = 77) unchanged. In other words, for each permutation sample, 34 HES subjects were randomly chosen from the original sample, allowing the same HES subject to be included multiple times. Similarly, 77 LES subjects were randomly selected from the original sample, allowing for replications of the selected subjects. This permutation sample thus contained the same numbers of HES subjects and LES subjects as in the original dataset, but represented a different inter-subject variability. Third, for each permutation sample, the difference in the average similarity degree between the HES subjects and the LES subjects was calculated and this procedure was repeated 6,000 times. Our null hypothesis was that there was no significant difference in the average similarity degree between the HES subjects and the LES subjects.

After permutation test, we found that there was a significant difference in the average similarity degree between the HES subjects and the LES subjects (*p* = 0.02). And through the visual inspection, we can find that the average similarity of HES subjects is higher (Figure 2B). The subjects within homogeneous communities have a common intrinsic connective network. Thus, it can be concluded that the activation of SN can distinguish the HES subjects from the LES subjects.

#### Analysis of voxel-wise functional connectivity strength

The gRAICAR results revealed that the inter-subject similarity reflected in the SN is associated with Extroversion classification. We then performed *post-hoc* functional connectivity analyses within the SN, aiming to investigate the strength of functional connectivity between the regions in the data-driven identified ICN. By this step, we could verify the association between the functional connectivity strength, measured using Pearson's correlation coefficient, and the Extroversion scores. Obviously, the analysis of this step was different from the gRAICAR analysis that attempted to associate large-scale networks patterns (obtained using ICA analysis) with personality inventory classifications and further provided supporting evidence for the findings from the gRAICAR analysis.

For the SN, we obtained three regions of interest (ROIs) by applying a threshold of *Z* > 2.5 and a cluster size > 30 voxels to its brain map, including the anterior cingulate cortex (ACC) and the bilateral insula. For each subject, the time series from each voxel within the ROIs were extracted to construct a voxel-wise functional connectivity matrix. The correlation coefficients between voxels belonging to different ROIs were converted into Fisher's *Z*-values and we obtained a matrix of inter-ROI functional connectivity (inter-FC) by averaging the functional connectivity matrices from individual subjects. A network model highlighting the functional connectivity difference between the HES and LES subjects is shown in Figure 2C and the corresponding statistical comparisons are displayed in Figure 2D. A linear model was used to examine the differences in inter-FC between HES and LES subjects, where age (centered to mean) and gender (coded as a factor) were included as covariates. The results showed higher inter-FC in HES subjects than in LES subjects in all three connections in Figure 2C (connection 1, *t* = −2.89, *p* = 0.004; connection 2, *t* = −2.48, *p* = 0.01; connection 3, *t* = −2.15, *p* = 0.03). These observations confirmed that LES subjects exhibit aberrant functional connectivity within the salience network.

#### Correlations between extraversion scores and connectivity strength in salience network

**Figure 3:**
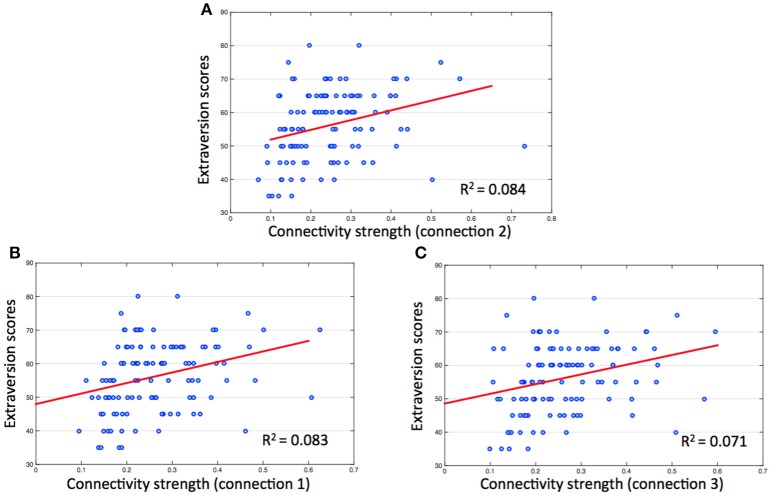
Correlations between extraversion scores and connectivity strength in salience network. The numbers on the y, x coordinate indicate extraversion scores and connectivity strength (Fisher's Z) between each ROI of salience network. **(A)** Connection 2 (between ACC and right insula), *r* = 0.304, *p* < 0.01; **(B)** connection 1 (between ACC and left insula), *r* = 0.302, *p* < 0.01; **(C)** connection 3 (between bilateral insula), *r* = 0.282, *p* < 0.01.

Results of the hierarchical regression indicated the best predictor of Extroversion scores was connectivity strength between the ACC and the right insula (Figure 3A). The predictor accounted for 8.4% of the model variance (*R*^2^adj = 0.084, *F* = 10.966, *p* < 0.01). The other two connectivity strengths (connection 1, *R*^2^adj = 0.083, *F* = 10.858, *p* < 0.01; connection 3, *R*^2^adj = 0.071, *F* = 9.341, *p* < 0.01) were also significant predictors of Extroversion scores (Figures 3B,C).

## Discussion

In the present study, following a new research strategy, we applied a systematic data-mining approach to investigate the characteristics of ICNs with a sample of 111 healthy subjects and found a reliable association between the salience network (SN) characteristic and EPQ-Extroversion classification. Specifically, there is a great possibility that people who have the high activation of SN will get a high score on the EPQ-Extroversion trait.

As one of the most important dimensions of personality traits, extraversion is the most stable core and a universal component in personality theory and it also is the best established and validated (Eysenck and Eysenck, 1994; Lei et al., 2015). Moreover, extraverts are typically described in positive emotional terms such as excitement, engagement, and enthusiasm (Lei et al., 2015). The salience network has been confirmed in the brain's intrinsic functional architecture and it can integrate a variety of external cognitive and emotional information, identifying the most significant stimuli, guiding the allocation of attention (Seeley et al., 2007; Menon and Uddin, 2010). Meanwhile, SN plays an important role as a “switch” between the self monitoring network and the task related network, that is, it plays a major role when the individual is in the process of the external information from “awareness” to “consciousness” (Palaniyappan and Liddle, 2012). The salience network is mainly composed of anterior cingulate and bilateral anterior insula (Menon, 2015), in fact, there are a large number of researchers who have studied the relationship between this three brain regions and extraversion as well as extraversion related personality traits by positron emission tomography (Johnson et al., 1999; Sugiura et al., 2000), task-fMRI (Kumari et al., 2004), rest-fMRI (Wei et al., 2011) and structural MRI (Van Schuerbeek et al., 2011). Previous studies have shown that the insula and cingulate gyrus are positively correlated with extraversion, which finding is consistent with the results of our study. In summary, prior work already related connectivity in the salience network with Extraversion, and the main contribution of this study is to replicate this finding with a “brain first” research strategy, and the new research strategy will be discussed in the next section.

### Research strategy angle: focusing on the difference of brain imaging characteristics

Many basic psychological concepts relying on the experience of the observer which situation is the obstacle of psychology as a mature science (Barrett, 2005, 2006, 2009). Since facing the same conundrum, researchers have begun to focus on exploring the biological basis of personality traits, mainly brain function or structural basis, thus trying to make more objective interpretations of the concepts of personality traits. The hypothesis of these related studies is that the concept itself of personality traits has an objective biological basis, however, the formation of the concept would be more or less affected by the personality inventory makers' life observations and experiences. From this perspective, the current researches on the brain mechanism of personality trait seems to be still unable to get rid of the influence of human subjective experience, thus affecting the objectivity of the research conclusions.

In the field of clinical psychiatric diagnosis, a series of achievements have been obtained in the studies (Fair et al., 2012, 2013; Yang et al., 2014a,b) that adopted the spirit of the “Research Domain Criteria” project (Insel et al., 2010; Insel, 2014; Simmons and Quinn, 2014) that establishing the mental disorders classification system based on brain mechanism. It is worth our attention, the new association between some of the brain functional networks and individual symptoms found in these studies is difficult to be found in the study based on the concept of psychology and semiology.

Accordingly, we decided not to adopt the traditional research strategy when we started to design this study, instead, the research strategy that we adopted in this study is to emphasize the establishment of the hypothesis based on the difference of inter-individual brain imaging characteristics, and then infer the personality traits associated with a certain ICN characteristic.

### Research method angle: methodological advantages of gRAICAR

Originated from people's life experience, psychological concept does not necessarily reflect the functional organization of the nervous system. Thus, consulting a variety of neuroimaging studies on psychological concept, we are confused that a brain area is related to many psychological concepts that have a great different definition (Smith et al., 2009; Zhang and Raichle, 2010; Gasquoine, 2013). Apparently, the research of personality brain mechanism also has this kind of confusion. Actually, with complex interactions from multiple dimensions of personality, neuroimaging data are usually heterogeneous. Findings obtained from the assumption of group homogeneity often unable be replicated (Miller, 2009). For addressing these problems, here, we presented a fully data-driven ICA-based method (gRAICAR) which does not assume that all subjects share the same set of components, meanwhile, it can provide a data-mining tool searching for the existence of heterogeneous subject communities according to functional neuroimaging data. Thus, gRAICAR provides a neuroimaging-based exploratory tool and makes it suitable for generating specific hypotheses for further examination.

### Limitations and future directions

There are three limitations exist in the current study. First, only one scale (EPQ) was used in this study, actually, as an exploratory work, we should adopt several different personality inventories including more personality traits in order to facilitate the establishment of associations between more ICNs and personality traits. Second, the differences in SN functional connectivity between high/low Extraversion subjects seems to be circular with the finding that HES subjects are more homogeneous in their spatial components compared with LES subjects, and that makes this experiment exploratory. Thus, in the future research, it is better to collect another validation sample to verify the exploratory findings. Besides, considering that repeatability is an important and normal part of science, we decided to share the data on Human Brain Data Sharing Initiative (HBDSI) (http://mrirc.psych.ac.cn/HBDSI) in the second half of 2018. Third, more and more studies have indicated that functional connectivity shows noticeable variations over a range of seconds to minutes, even in the resting state without external stimuli. Therefore, it is necessary for future researchers to explore the dynamic functional connection characteristics of different personality traits.

## Author contributions

XM: designed and supervised the study. FT, JW, and CX: drafted the manuscript. FT: carried out the experimental procedures. JW, CX, and HL: participated in data processing.

### Conflict of interest statement

The authors declare that the research was conducted in the absence of any commercial or financial relationships that could be construed as a potential conflict of interest.
