# Corrigendum: Focusing on the Differences of Resting-State Brain Networks, Using a Data-Driven Approach to Explore the Functional Neuroimaging Characteristics of Extraversion Trait

**DOI:** 10.3389/fnins.2018.00380

**Published:** 2018-05-31

**Authors:** Feng Tian, Junjie Wang, Cheng Xu, Hong Li, Xin Ma

**Affiliations:** ^1^Beijing Anding Hospital of Capital Medical University, Beijing, China; ^2^Department of Psychiatry, The Second Hospital of Shanxi Medical University, Taiyuan, China; ^3^Department of Psychiatry, First Hospital/First Clinical Medical College of Shanxi Medical University, Taiyuan, China; ^4^Department of Magnetic Resonance Imaging, Shanxi Province People's Hospital, Taiyuan, China; ^5^Institute of Psychology, Chinese Academy of Sciences, Beijing, China

**Keywords:** personality traits, resting-state fMRI, data-driven, salience network, extraversion trait

Incorrect Affiliation of first author:

1 Beijing Anding Hospital of Capital Medical University, Beijing, China

2 Department of Psychiatry, The Second Hospital of Shanxi Medical University, Taiyuan, China

In the published article, there was an error regarding the affiliation**s** for **Feng Tian**. As well as having Affiliation **1**, they should also have **Beijing Anding Hospital of Capital Medical University, Beijing, China**. Meanwhile, Beijing Anding Hospital of Capital Medical University as the first affiliation is vital. Department of Psychiatry, The Second Hospital of Shanxi Medical University, Taiyuan, China must be the second affiliation.

The authors apologize for this error and state that this does not change the scientific conclusions of the article in any way.

The original article has been updated.

## Conflict of interest statement

The authors declare that the research was conducted in the absence of any commercial or financial relationships that could be construed as a potential conflict of interest.

